# The Danger of False Teeth

**Published:** 1897-01-23

**Authors:** 


### THE DANGER OF FALSE TEETH.

A curious case was mentioned at a recent meeting of the Pathological Society. A plate holding an artificial tooth had been swallowed eight months before, the accident having happened during a faint which occurred while the patient was out shopping. On a medical man passing a probang he felt a sensation as if an obstructing body had been pushed forward into the stomach. After this the patient had comparatively little discomfort and no difficulty in swallowing unless she took something solid in rather larger mass than usual, when she sometimes had a suffocative sensation for a time. She was pregnant when the accident happened. After the confinement she had pain in the abdomen and in the iliac fossa, so an attempt was made to ascertain the position of the foreign body by means of the *x* rays. For this purpose the abdomen was skiagraphed, but without result. As the plate which had been swallowed never came away she went into St. Thomas's Hospital with a view to operative measures, but discharged herself before anything was done. Some time after this she suddenly vomited a large quantity of blood. Again an attempt was made to fix the position of the plate, and another skiagraph of the abdomen was taken; the bleeding, however, continued, and she died; in fact, she died while the skiagraph was being taken. But on post-mortem it was found that the denture had never entered the stomach at all. It had become buried in the tissue outside the œsophagus just below the bifurcation of the trachea, and had ulcerated its way into the thoracic aorta. The plate was a small one, carrying one tooth.

